# Case Report: A Chinese Family of Hypertrophic Cardiomyopathy Caused by a Novel Splicing Mutation in the *FLNC* Gene

**DOI:** 10.3389/fgene.2022.894791

**Published:** 2022-06-20

**Authors:** Chunhui Huang, Yonghong Zheng, Wei Zhang, Zhigang Chen, Zhixin Huang, Yuan Fang

**Affiliations:** ^1^ Department of Cardiology, Liyang City Hospital of Traditional Chinese Medicine, Liyang, China; ^2^ Department of Ultrasound Medicine, Liyang City Hospital of Traditional Chinese Medicine, Liyang, China; ^3^ Department of Geriatric Medicine, Affiliated Hospital of Nanjing University of Chinese Medicine, Nanjing, China

**Keywords:** cardiomyopathy, hypertrophic, *FLNC*, splicing mutation, exome sequencing

## Abstract

Hypertrophic cardiomyopathy (HCM) is a type of primary cardiomyopathy with genetic etiology, and it carries a high risk of diastolic dysfunction, heart failure, and malignant arrhythmias. We reported the first familial HCM in China, caused by a novel *FLNC* splicing mutation. We performed duo exome sequencing (ES) to examine the genome of the proband and his mother. For 10 days, a 15-year-old boy was presented to our hospital due to non–exercise-associated chest tightness and asthma. He was diagnosed with HCM [end-diastolic interventricular septal thickness was about 18 mm by transthoracic echocardiography (TTE)]. His mother and sister performed TTE to screen familial cardiomyopathy, which revealed hypertrophic cardiomyopathy only in the proband’s mother. In ES of the mother–son duo, we identified a novel heterozygous mutation of the *FLNC* gene (chr7:128492808, NM_001127487, c.5905+2T>C, rs1808874360) as the candidate cause of autosomal dominant HCM. Sanger sequencing confirmed this novel mutation in the proband and his mother but absent in the proband’s sister. The potential impact of the novel mutation was predicted by MutationTaster, dbscSNV_ADA_SCORE, dbscSNV_RF_SCORE, CADD_phred, PhyloP20way_mammalian, PhyloP100way_vertebrate, SiPhy_29way_logOdds, and GERP++_RS software. After the administration of furosemide, spironolactone, and metoprolol, the proband’s heart function was improved, and symptoms were alleviated. We presented the first familial HCM caused by a novel *FLNC* splicing mutation *via* exome sequencing in China. Therefore, it is necessary that familial screening for patients with HCM should be performed for the early detection of HCM intervention in malignant cardiac events in advance and block genes.

## Introduction

Hypertrophic cardiomyopathy (HCM) is one of the most typical causes of inherited heart disease. Most data showed an estimated prevalence of 0.5% in the past 20 years (Semsarian et al., 2015). Pathophysiologically, all HCM complications may relate to the existence and evolution of fibrosis (Pagourelias et al., 2021), which contributes to increased mechanical stiffness, leading to diastolic dysfunction, the transition toward heart failure, and the development of cardiac arrhythmias (Fujita et al., 2015; Disertori et al., 2017; O’hanlon et al., 2010). Various morphological variants of HCM are known and present with different hemodynamic and clinical manifestations, resulting in ischemia of the myocardial tissue, mitral regurgitation, left ventricular outflow tract obstruction, and stiffening of the left ventricle. Meanwhile, these changes can cause typical symptoms of dyspnea, chest pain, palpitations, and syncope (Veselka et al., 2017). HCM is a genetic (autosomal dominant) heart muscle disease caused by a mutation in the sarcomere protein genes which encodes for elements of the contractile machinery of the heart (Basit et al., 2021). Over 1,500 mutations in at least 15 sarcomere-encoding genes have been identified (Ingles et al., 2015). HCM represents the most common monogenic cardiomyopathy in humans (Tuohy et al., 2020). Filamin C (*FLNC*) is a protein-coding gene. *FLNC* is an essential structural crosslinker of actin rods at the sarcomeric z-disc of cardiac and skeletal muscle (Verdonschot et al., 2020). Initially, mutations in *FLNC* were only linked to myofibrillar myopathy (MFM) but are now increasingly found in various forms of human cardiomyopathy (Eden and FREY, 2021). This study described a familial HCM with a novel *FLNC* mutation identified by exome sequencing (ES).

## Case Presentation

**FIGURE 1:**
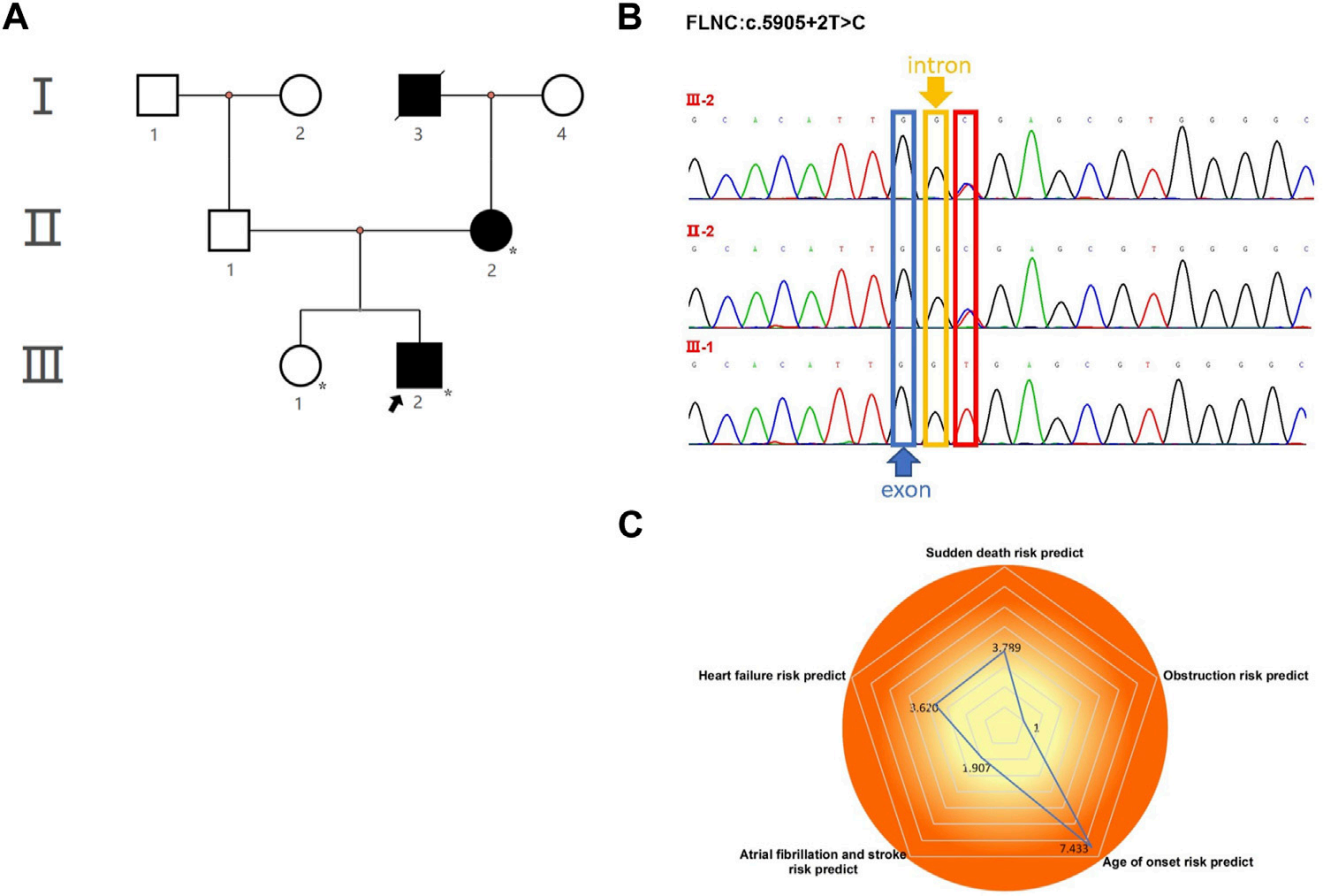
Pedigree analysis and sequencing results. **(A)** Pedigree of the proband (arrow) and his family members with heterozygous *FLNC* mutations. The star symbol indicates the used WES. **(B)** Sanger sequencing confirmed a splicing mutation in *FLNC*, which was of maternal origin. It is highlighted in the red box. **(C)** Assessment of five prognostic risk factors for HCM.

**FIGURE 2:**
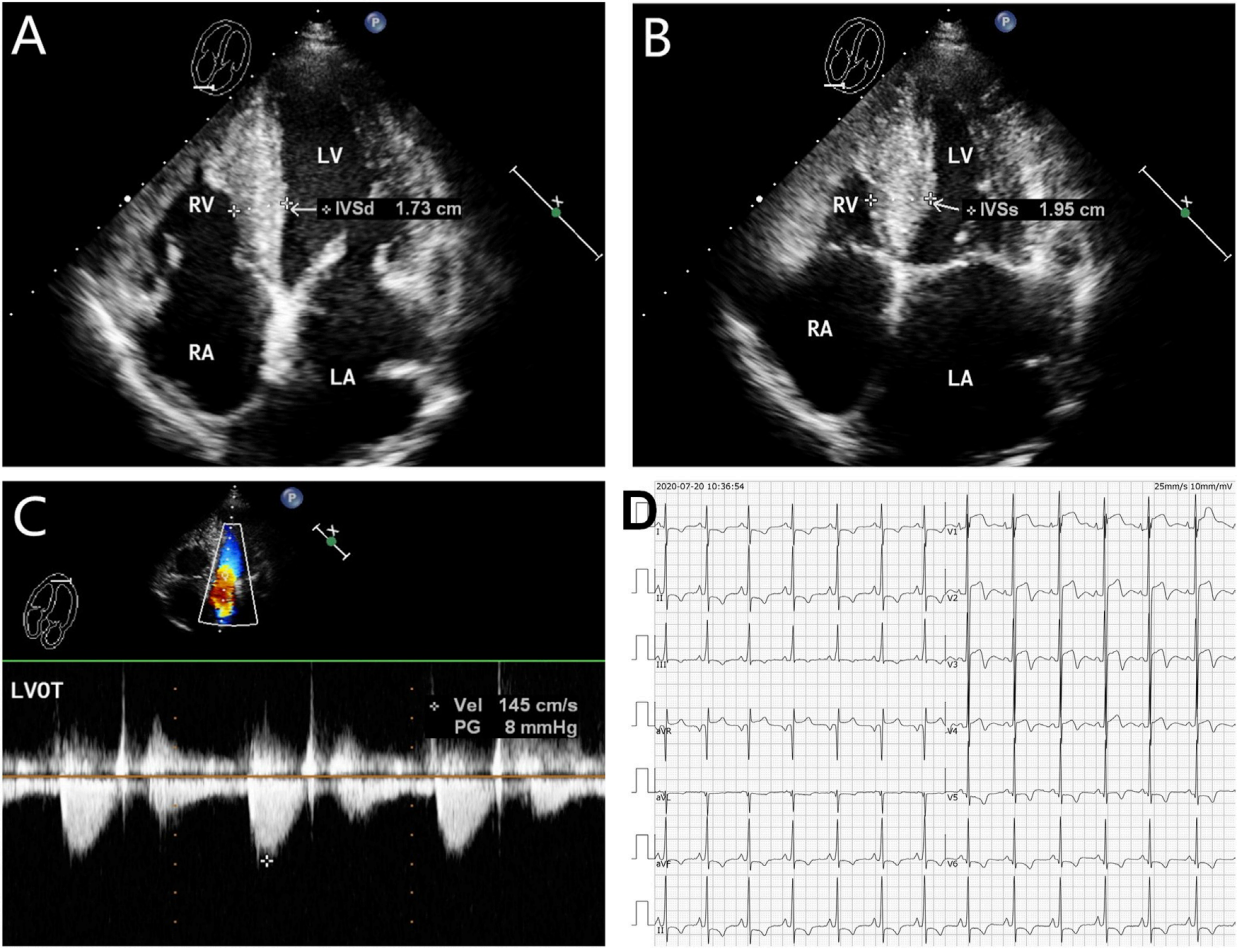
Echocardiography and electrocardiography in the proband (**III-2**). **(A–C)** Echocardiography and color Doppler showed enlargement of the left atrium, thickening of the interventricular septum at the end-diastole, and no outflow obstruction, EF 67%. **(D)** ECG showed that inferior and high lateral wall leads to ST-segment depression with T-wave inversion; right precordial leads to ST-segment elevation.

**FIGURE 3:**
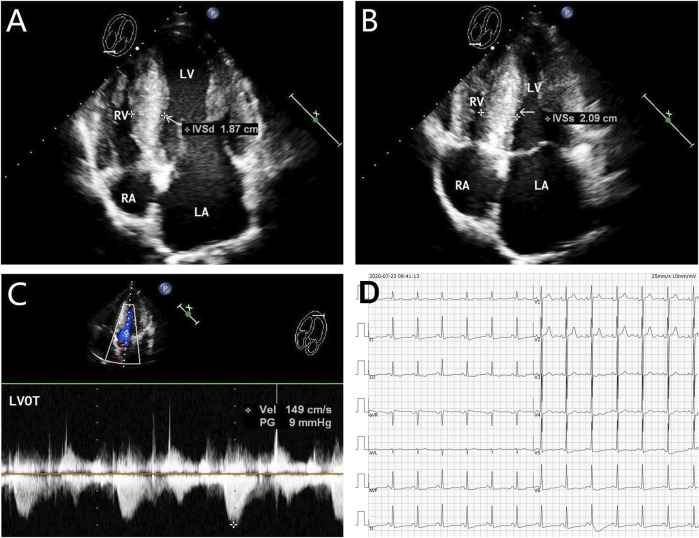
Echocardiography and electrocardiography in the proband’s mother (**II-2**). **(A–C)** Echocardiography and color Doppler showed enlargement of the left atrium, thickening of the interventricular septum at the end-diastole, and no outflow obstruction, EF 62%. **(D)** ECG showed that inferior and high lateral wall leads to ST-segment depression, T-wave low flat, or T-wave inversion.

The proband (**III-2** in Figure 1A) was a 15-year-old male from Chinese. He was presented to our hospital due to non–exercise-associated chest tightness and asthma for 10 days. The abovementioned symptoms last a few minutes every time. There was no history of tobacco, alcohol, or recreational/illicit drug use. The physical examination results showed that the vital signs were typical, forceful apex beat, and cardiac enlargement could be palpated. His electrocardiogram showed ST-segment depression in I, II, aVF, aVL, and V5-V6 leads, ST-segment elevation in V1-V4 leads, and T-wave inversion in II, aVF, and V2-V6 leads, QT interval prolongation, left atrial enlargement, and R/S ratio > 1 in V1 and V2 leads (Figure 2D). A transthoracic echocardiogram showed that the end-diastolic interventricular septal thickness was about 18mm, left atrium enlargement, left atrial dimension (LAD) was about 43 mm, and mild mitral and tricuspid regurgitation (Figures 2A–C, Table 1). The level of serum NT-proBNP was elevated at 1,001 pg/ml. The level of serum creatine kinase was normal, according to 2020 AHA/ACC Guideline recommendations for managing patients with nonobstructive HCM with preserved EF. The patient received diuretics (furosemide 20 mg QD and spironolactone 20 mg QD) and metoprolol 47.5 mg QD to improve the heart function. After 4 days of treatment, chest tightness and asthma symptoms were alleviated. As inherited cardiomyopathy, clinical screening of the proband’s family is also necessary. The proband’s grandfather (**I-3** in Figure 1A) died at 72 years due to cardiac disease. The proband’s grandmother (**I-4** in Figure 1A) did not have any cardiac disease. The proband’s father (**II-1** in Figure 1A) did not present any cardiac symptoms and was not investigated. The proband’s mother (**II-2** in Figure 1A) had a medical history of cardiac disease. She was admitted to another hospital previously because of chest tightness. In this investigation, her electrocardiogram showed ST-segment depression in I, II, III, aVF, and V3-V6 leads, T-wave low flat in I, II, avF, and V3-V6 leads, with T-wave inversion in III lead (Figure 3D). Her transthoracic echocardiogram showed that the end-diastolic interventricular septal thickness was about 17 mm, left atrium enlargement, mild mitral and tricuspid regurgitation, and pulmonary artery systolic pressure about 36 mmHg (Figures 3A–C, Table 1). The proband’s sister (**III-1** in Figure 1A) was utterly normal from birth to adolescence and showed no cardiac symptoms, and her transthoracic echocardiogram showed normal cardiac function and geometry. His symptoms were stable during a year of followup. For familial reasons, the patient refused the electrocardiogram and echocardiography examination after leaving the hospital.

**TABLE 1:** Clinical characteristics of individuals harboring the novel *FLNC* mutation.

Characteristic	Mother	Proband	Sister
	II-2	III-2	III-1
Sex/Age (year)	Female/48	Male/15	Female/22
Electrocardiogram	Sinus rhythm	Sinus rhythm	Normal
	ST-segment depression	ST-segment change	
	T-wave low flat or T-wave inversion	T-wave inversion	
Transthoracic echocardiography	HCM	HCM	Normal
	LA 49 mm	LA 43 mm	LA 29 mm
	IVSd 17 mm	IVSd 18 mm	IVSd 7 mm
	EF 62%	EF 67%	EF 63%
	FS 34%	FS 37%	FS 34%

## Exome Sequencing of the Mother–Son Duo

The procedures used in this study conformed to the tenets of the Declaration of Helsinki. The studies involving human participants were reviewed and approved by the ethics committee of Liyang City Hospital of Traditional Chinese Medicine. Written informed consent to participate in this study was provided by the participant’s legal guardian/next of kin.

The exomic DNA of the proband (**III-2**), the proband’s mother (**II-2**), and his sister (**III-1**) were used in Agilent’s SureSelect Human All Exon V6 (Agilent Technologies, Santa Clara, CA, United States). The V6 panel was used for the whole-exome capture with DNA samples, and enriched DNA from exome capture was used to construct libraries. Whole-exome sequencing (WES) was performed on the Illumina platform (U.S.), following a quality inspection. After acquiring the raw sequenced reads, bioinformatics analysis was completed in conjunction with the reference or genome sequence.

The HCM-related genes were assembled from the databases: genes associated with the relationships between variation and human health from ClinVar (http://www.ncbi.nlm.nih.gov/ClinVar) and OMIM (http://www.omim.org). Nuclear genes underlying or associated with human inherited disease in HGMD (http://www.hgmd.org). The HCM-related genes were filtered by the population sequencing database: Exome Aggregation Consortium (ExAC), 1,000 Genomes Project, Exome Variant Server (ESP6500), and Genome Aggregation Database (gnomAD). The pathogenicity classification was interpreted according to American College of Medical Genetics and Genomics (ACMG) guidelines (Richards et al., 2015).

The potential risk predictor model (Nuoqingxin^®^) was developed by Bestnovo (Beijing) Medical Laboratory Co., Ltd., which by adopting the world’s advanced gene sequencing technology to detect the hypertrophic cardiomyopathy genes combined with the risk assessment system of the AHA guideline, the sudden cardiac death prediction model of the ESC guideline, and with the Chinese population hypertrophic cardiomyopathy gene database extension and supplement. It could conduct a comprehensive individualized risk stratification, prognosis assessment, and intervention guidance.

## Functional Prediction of the Mutation

Computational prediction tools (MutationTaster, CADD_phred, PhyloP20way_mammalian, PhyloP100way_vertebrate, SiPhy_29way_logOdds, and GERP++_RS) were used to predict the conservation and pathogenicity of mutation. Splice-site prediction software programs were selected for this study. Two ensemble prediction scores (dbscSNV_ADA_SCORE and dbscSNV_RF_SCORE), using adaptive boosting and random forest ensemble learning methods, were extracted from the dbscSNV database.

## Result

By estimating the sequence quality, 818 million reads were generated from the proband’s sample. The percentage bases above average ×10 were achieved for 96.43%, and the mean read depth (×) was 96.78 and a Q30 value of 99.21%.

ES of the mother–son duo identified two heterozygous variants as candidate causes of autosomal dominant inherited diseases. The *FLNC* mutation seemed pathogenic, and the *SCN5A* mutation was reported to be likely pathogenic (Supplementary Table S1).

In the proband and his mother presenting with a similar phenotype, the *FLNC* mutation (chr7:128492808, NM_001127487, c.5905+2T>C, rs1808874360) was located in exon 35. To confirm the presence of *FLNC* c.5905+2T>C mutation in the affected members, we performed Sanger sequencing. It proved the heterozygous c.5905+2T>C variant in the proband and his mother, while it was absent from the blood DNA of the proband’s sister (Figure 1B). Thus, the variant identified in the affected individuals was demonstrated to cause autosomal dominant cardiomyopathy. Human Splicing Finder software predicted the alteration of the WT Donor site in the *FLNC* gene, most probably affecting splicing. This splicing mutation of the *FLNC* was expected to be “likely pathogenic” in the ClinVar database or “disease-causing mutation” in the HGMD database by the computational *in silico* prediction. By September 2021, clinical significance and variant classification were performed in the ClinVar database. A total of 1,113 missense variants in *FLNC* were represented, of which 15 were classified as pathogenic and/or likely pathogenic (1.35%). However, 34 of 35 splicing mutations were classified as pathogenic and/or likely pathogenic (97.14%). The annotation in the ClinVar database is not specific for a given phenotype. For individuals with pathogenic or likely pathogenic variants linked to *FLNC*, we detected that 14 of 180 were missense variants, and 34 of 180 were splicing mutations. The *FLNC* (c.5905+2T>C) mutation was not in the protein functional domain by checking the interpro database. The *FLNC* heterozygous variant was absent in the database Exome Aggregation Consortium (EXAC, http://exac.broadinstitute.org/), 1,000 Genomes Project (http://browser.1000genomes.org), Exome Variant Server (ESP6500, https://evs.gs.washington.edu/EVS/), and the Genome Aggregation Database (GnomAD, http://gnomad.broadinstitute.org/), indicating that it was a new candidate disease-causing gene. According to several different types of bioinformatics software, the mutation was predicted to be disease-causing in MutationTaster, consistent with the score of, and the high CADD_phred score of 24.3 also suggested a harmful effect of the mutation (Supplementary Table S2). The dbscSNV_ADA_SCORE and dbscSNV_RF_SCORE were 1.0 and 0.93, respectively, which indicated to affect splicing (Supplementary Table S2). The prediction tools for conservation thresholds for phyloP20way_mammalian, PhyloP100way_vertebrate, SiPhy_29way_logOdds, and GERP++_RS were 1.058, 8.017, 16.450, and 4, respectively (Supplementary Table S3). Moreover, the mutation has been classified as pathogenic (PVS1, PM2, and PP1) based on the American College of Medical Genetics (http://wintervar.wglab.org) guidelines.

In the present study, we used the Nuoqingxin^®^ risk model to evaluate the five prognostic risk factors of the HCM patient. We found that the age of the onset risk index was 7.433, the probability of early-onset (before 40 years of age) was about 60%, and the most likely age of onset was between 24.15 and 35.29 years old. The risk index of the left ventricular outflow tract obstruction was 1, and the possibility of obstruction was low, about 26.95%. If an obstruction occurred, the average age of onset was approximately 54.51 years old. The risk index of sudden cardiac death was 3.789. The risk of sudden cardiac death was high, with a total probability of 34.29%. The average time for malignant arrhythmia and even sudden cardiac death was about 11.47 years after hypertrophic cardiomyopathy. The heart failure risk index was 3.620, the probability of occurrence was approximately 39.32%, and the average age of the event was about 51.90 years old. The risk index of atrial fibrillation and stroke was about 1.907, the probability of occurrence was about 25%, and the average age of the event was approximately 57.64 years old.

## Discussion

HCM represents one of the primary cardiomyopathies and can present with the symptoms at any phase of life. It is a myocardial disorder characterized by left ventricular hypertrophy (LVH) and not explained by other causes of hypertrophy conditions such as hypertension, congenital heart disease, and metabolic diseases associated with myocardial hypertrophy. Due to clinical heterogeneity, many individuals present heart failure, atrial fibrillation, or sudden cardiac death, while others can have minimal or no symptoms. Routinely, the diagnosis of HCM relies on noninvasive imaging studies, such as transthoracic echocardiography and cardiac magnetic resonance imaging. In addition to imaging studies, genetic testing can help illustrate the underlying genetic cause of the disease. In recent years, numerous reports from the literature of causative variants in cases with HCM provide genetic evidence. Those classified as definitive included well-known disease genes that have been included in eight well-established sarcomeric genes (*MYBPC3*, *MYH7*, *TNNT2*, *TNNI3*, *TPM1*, *ACTC1*, *MYL3*, and *MYL2*) (Ingles et al., 2019). Furthermore, in rare cases, mutations in genes encoding for proteins involved in the Ca^2+^-homeostasis like PLN, or genes encoding for Z-disc proteins, like ACTN2 or *FLNC*, are also known to cause HCM (Brodehl et al., 2019). *FLNC* is a structural protein, with the primary role in maintaining the structural integrity of the sarcomere. This is through the crosslinking actin filaments and the anchoring of sarcolemmal proteins to the cytoskeleton (Verdonschot et al., 2020). Among its related pathways are cell junction organization and cytoskeletal signaling. Variants in *FLNC* are traditionally associated with MFM, but subsequently also with isolated cardiomyopathies. High‐throughput sequencing studies of *FLNC* were more frequently used in the cardiomyopathy cohorts because more prevalent was this clinical entity, and more genetic variants were described in cardiomyopathy patients than myopathies. From the time that *FLNC* was recognized as a disease‐associated gene in the field of cardiomyopathies, *FLNC* mutations have been related to dilated cardiomyopathy (DCM) (Verdonschot et al., 2020), hypertrophic cardiomyopathy, and other cardiac phenotypes such as arrhythmias without detectable structural abnormalities, congenital heart disease, restrictive cardiomyopathy (RCM) (Brodehl et al., 2016), and noncompaction cardiomyopathies (NCCM) (Ader et al., 2019). The previous study found that HCM was mainly associated with missense variants (Gómez et al., 2017; Ader et al., 2019), which causes changes in the secondary protein structure, resulting in an abnormal protein. Abnormal protein has been observed within aggregates in the tissue of *FLNC*‐associated HCM patients associated with marked sarcomeric abnormalities (Valdés-Mas et al., 2014). The progressive accumulation of protein aggregates in the cardiac muscle eventually leads to sarcomeric disarray. Genetic investigations confirmed that our patients have HCM due to a classified as-likely pathogenic variant or disease-causing mutation, predicted to alter the WT donor site in the *FLNC* gene, most probably affecting splicing. The mutation was predicted to be disease-causing by MutationTaster. Moreover, the bioinformatics program (PhyloP20way_mammalian, PhyloP100way_vertebrate, SiPhy_29way_logOdds, and GERP++RS) revealed this mutation site to be evolutionarily highly conserved. In the absence of left ventricular outflow tract obstruction, we treated the patient with improving heart failure symptoms, recommending advanced treatment options (e.g., cardiac resynchronization therapy, left ventricular assist device, and transplantation). The patient’s symptoms were alleviated. It was worth mentioning that genetic investigations also reported the proband to carry mutation in *SCN5A*. *SCN5A* mutations were the first genetic variants shown to be associated with Brugada syndrome, both loss- and gain-of-function mutations, which may cause dilated cardiomyopathy (Wilde and AMIN, 2018). We performed the cross-prediction of *SCN5A* mutations with multiple kinds of bioinformatic prediction software (including SIFT and Polyphen-2), and the results were mostly deleterious. The ClinVar database was consulted, and it was found that the mutation was rated as “Uncertain_significance” by the reporter, and the HGMD database did not find the mutation. According to the available evidence, the mutation is rare, the software application predicts that the mutation may have an impact on protein function, and the amino acid at this position is well conserved in vertebrates, but one in the local database control population carries the mutation, and one who carries this variant cannot determine whether they have related phenotypes, lack of family linkage, and functional evidence support. This mutation is considered a suspected pathogenic variant. The proband presented non–exercise-associated chest tightness and shortness of breath. The echocardiogram showed that the thickness of the interventricular septum was increased. Therefore, there was no sufficient evidence that *SCN5A* variants are associated with the symptoms in the proband. To date, the cases in our study were the first cases that reported familial HCM caused by *FLNC* splicing mutation and were identified by a duo ES approach to examine the genome of the proband, proband’s sister, and proband’s mother in China. Notably, *FLNC* splicing mutation has rarely been reported among families with HCM. However, splicing mutations seem to be more pathogenic. The *FLNC* mutation may lead to cardiomyopathies and myopathies, as we mentioned previously. In our study, ECG and TTE of the proband and his mother revealed thickening of the left ventricular septum results. On the other hand, in a previous cohort of patients with HCM, it was reported that 34% of the *FLNC* mutation carriers had elevated creatine kinase (CK) levels (Valdés-Mas et al., 2014). *FLNC,* as a sarcomere mutation, is related to MFM and distal myopathy (Kley et al., 2012; Kley et al., 2013). Accordingly, we cannot exclude the occurrence of distal myopathy someday in our affected individuals with the *FLNC*-related cardiomyopathy. 2020 AHA/ACC Guideline recommended genetic testing to elucidate the genetic basis to facilitate the identification of family members at a risk for developing HCM (cascade testing) (Ommen et al., 2020). Because the father line did not affect, the mutation was inherited from the mother. The first-degree relatives (especially the maternal line), clinical screening (ECG and TTE), and cascade genetic testing should be offered to confirm the further co-segregation mutation in the family. The proband’s sister’s genotype is negative, showing that continuous clinical screening is not indicated. Genetic testing predicts adverse clinical outcomes and becomes an essential part of guiding risk stratification as genetics grows. In the future, genotype may play a more significant role in risk stratification, management, treatment, and prognosis, offering improved outcomes for patients and their families with HCM (Stafford et al., 2021). A recent study proposes that rare variants associated with the inherited arrhythmic syndrome should be reanalyzed within 5 years, if already classified following ACMG recommendations, since it seems to be adequate to manage the rapid obsolescence of genetic data interpretations (Campuzano et al., 2020). Genetic testing is an integral part of clinical practice as it carries both the diagnostic and prognostic value. It should be noted that the correct diagnosis of hypertrophic cardiomyopathy requires the patient’s symptoms combination with advanced imaging techniques. Misinterpretation of rare variant designations may lead to inaccurate genetic diagnoses and/or the adoption of unnecessary and/or inappropriate therapeutic approaches, resulting in an increased morbidity and mortality (Campuzano et al., 2020). Therefore, discriminating a true risk-carrying variant from a non-deleterious variant is a challenge. Further work may determine mutations identified in genes with robust gene–disease associations that could assist clinicians with the decisions regarding management and therapies in high-risk individuals.

In conclusion, we described a Chinese family with *FLNC* rare splicing mutation associated with HCM. Genetic testing clarified the carrying status of the mutation detected by the family members. The pathogenic mutation could be described according to the clinical phenotype and the carrying status. Thus, genetic screening of the family for congenital cardiomyopathy is essential for patients with HCM. It is necessary to carry out a risk assessment for the carriers, aim to intervene malignant cardiac events in advance, and block genes.

## Data Availability

All datasets generated for this study are included in the article/Supplementary Material, further inquiries can bedirected to the corresponding author.
