# Supplementary material for: Case Report: A Chinese Family of Hypertrophic Cardiomyopathy Caused by a Novel Splicing Mutation in the FLNC Gene
Source: Front Genet. 2022 Jun 20;13:894791. doi: 10.3389/fgene.2022.894791 (PMC9251305; doi:10.3389/fgene.2022.894791)
Supplement: Supplementary file 3 [file Table1.doc]

**Supplement Table 1 Other pathogenic/likely pathogenic mutation identified**

| **Gene** | **Transcript** | **Exon** | **NAChange** | **AAChange** | **Heterogeneity** | **Pathogenicity** |
| --- | --- | --- | --- | --- | --- | --- |
| SCN5A | NM_000335 | exon17 | c.3133G>A | p.Val1045Met | heterozygosis | likely pathogenic |

**Supplement Table 2 Bioinformatic predict pathogenicity of the novel *FLNC* mutation**

| **MutationTaster** | **dbscSNV_ADA_SCORE** | **dbscSNV_RF_SCORE** | **CADD_phred** |
| --- | --- | --- | --- |
| 1^$^ | 1.0 | 0.93 | 24.3 |

^$^Disease causing

**Supplement Table 3 Bioinformatic predict conservation of the novel *FLNC* mutation**

| **PhyloP20way_mammalian** | **PhyloP100way_vertebrate** | **SiPhy_29way_logOdds** | **GERP++_RS** |
| --- | --- | --- | --- |
| 1.058^*^ | 8.017^*^ | 16.450^*^ | 4^*#^ |

^*^Higher values are more deleterious; ^#^Highly conservative

**Supplement Table 4 Timeline of medication**

| **Time** | **Names** |
| --- | --- |
| **Day 0-4** | Furosemide 20mg QD  Spironolactone 20mg QD  Metoprolol 47.5mg QD |
| **Day 5-Now** | Metoprolol 47.5mg QD |
